# Multimodal opioid-sparing pain management for emergent cesarean delivery under general anesthesia: a quality improvement project

**DOI:** 10.1186/s12871-022-01780-9

**Published:** 2022-07-27

**Authors:** Kelechi B. Anyaehie, Elaine Duryea, Jenny Wang, Chinedu Echebelem, Devin Macias, Mary Sunna, Olutoyosi Ogunkua, Girish P. Joshi, Irina Gasanova

**Affiliations:** 1 Department of Anesthesiology and Pain Management, UT Southwestern Medical Center, 5323 Harry Hines Blvd, Dallas, TX 75390-9068 USA; 2 Department of Obstetrics and Gynecology, UT Southwestern Medical Center, 5323 Harry Hines Blvd, Dallas, TX 75390-9159 USA; 3 Parkland Health and Hospital System, 5200 Harry Hines Blvd, Dallas, TX 75235 USA

**Keywords:** Cesarean delivery, Emergency surgery, Multimodal analgesia, Opioid-sparing, Postoperative pain

## Abstract

**Background:**

Opioid-sparing multimodal analgesic approach has been shown to provide effective postoperative pain relief and reduce postoperative opioid consumption and opioid-associated adverse effects. While many studies have evaluated analgesic strategies for elective cesarean delivery, few studies have investigated analgesic approaches in emergent cesarean deliveries under general anesthesia. The primary aim of this quality improvement project is to evaluate opioid consumption with the use of a multimodal opioid-sparing pain management pathway in patients undergoing emergent cesarean delivery under general anesthesia.

**Methods:**

Seventy-two women (age > 16 years) undergoing emergent cesarean delivery under general anesthesia before (*n* = 36) and after (*n* = 36) implementation of a multimodal opioid-sparing pain management pathway were included. All patients received a standardized general anesthetic. Prior to implementation of the pathway, postoperative pain management was primarily limited to intravenous patient-controlled opioid administration. The new multimodal pathway included scheduled acetaminophen and non-steroidal anti-inflammatory medications and ultrasound-guided classic lateral transversus abdominis plane blocks with postoperative opioids reserved only for rescue analgesia. Data obtained from electronic records included demographics, intraoperative opioid use, and pain scores and opioid consumption upon arrival to the recovery room, at 2, 6, 12, 24, 48, and 72 h postoperatively.

**Results:**

Patients receiving multimodal opioid sparing analgesia (AFTER group) had lower opioid use for 72 h, postoperatively. Only 2 of the 36 patients (5.6%) in the AFTER group required intravenous opioids through patient-controlled analgesia while 30 out of 36 patients (83.3%) in the BEFORE group required intravenous opioids.

**Conclusions:**

Multimodal opioid-sparing analgesia is associated with reduced postoperative opioid consumption after emergent cesarean delivery.

## Introduction

Cesarean delivery is one of the most commonly performed surgical procedures. In 2014, cesarean deliveries accounted for 32% of all births in the United States [1]. Post-operative pain after cesarean delivery can range from moderate to severe [2]. Inadequate pain management can interfere with maternal/newborn bonding, breastfeeding, mobilization and has been associated with postpartum depression, delayed recovery, chronic pain, and persistent opioid use [3, 4]. Nevertheless, pain after cesarean delivery continues to be inadequately treated probably due to fears that medications or interventions could negatively impact maternal or infant wellbeing and/or underestimation of pain severity [2].

Recently, the Procedure Specific Pain Management (PROSPECT) group published guidelines for pain management after elective cesarean delivery performed under neuraxial anesthesia [2]. Pain management for emergent cesarean delivery under general anesthesia was not addressed because of sparse evidence [2, 5]. Possible postoperative analgesic options for patients undergoing emergent cesarean delivery include epidural analgesia, if the patient has an indwelling epidural catheter and/or opioids. In the absence of an epidural catheter, multimodal opioid-sparing analgesic techniques have been recommended [2, 4]. These strategies include the use of acetaminophen, non-steroidal anti-inflammatory drugs (NSAIDs), dexamethasone, and local/regional techniques such as transversus abdominis plane (TAP) blocks [2–4]. This quality improvement project was designed to evaluate the analgesic efficacy of a multimodal opioid-sparing pain management pathway in patients undergoing emergent cesarean delivery under general anesthesia. The primary aim of this study was to evaluate opioid consumption at 48 h after surgery.

## Methods

This quality improvement project was performed at Parkland Health and Hospital Systems, Dallas, Texas, after approval from the Institutional Review Board (IRB) of the University of Texas Southwestern Medical Center, Dallas, Texas. A waiver for informed consent was obtained because deidentified patient data were collected from an electronic database.

The surgical cohort spanned from January 1 through December 31 of 2020. Consecutive patients (age > 16 years) undergoing emergent cesarean delivery under general anesthesia without an epidural before (BEFORE-Group, *n* = 36) and after (AFTER-Group, *n* = 36) implementation of a multimodal opioid-sparing pain management pathway were included. Exclusion criteria included an American Society of Anesthesiologists (ASA) physical status of ≥ 4, history of allergy to local anesthetics, concomitant neuraxial blocks in place, preoperative chronic opioid dependence or history of substance abuse, significant coagulopathies, infection or abnormalities at the TAP block injection sites.

All patients received a standardized general endotracheal anesthetic technique. This included induction with propofol (1–1.5 mg/kg, intravenous (IV)) and succinylcholine (1 mg/kg, IV). Maintenance of general anesthesia included nitrous oxide 50% in oxygen and desflurane or sevoflurane. Fentanyl 25–50 µg, IV, boluses were administered to maintain mean arterial blood pressure and/or heart rate within 20% of baseline values. Fentanyl was limited to less than 1 mcg/kg, IV, ideal body weight/hour. Prophylaxis for nausea and vomiting included dexamethasone 8 mg, IV, administered after induction of anesthesia, and ondansetron 4 mg, IV, administrated at the end of the procedure. Hydromorphone 0.2–0.4 mg, IV, was administered, as needed, approximately 20 min prior to the end of the procedure.

Prior to the introduction of a multimodal opioid-sparing pain management pathway, patients received morphine via intravenous patient-controlled analgesia (IV-PCA) with inconsistent use of acetaminophen or NSAIDs. As a part of the multimodal opioid-sparing pain management pathway, patients received bilateral ultrasound-guided TAP blocks performed using the classic lateral approach, by an anesthesia attending with significant experience in regional anesthesia at the end of surgery, after skin closure, but before emergence from anesthesia. An ultrasound transducer (linear 6–13 MHz, SonoSite M-Turbo, Brothell, WA) was placed transversely on the flank between the anterior superior iliac spine and the costal margin. Using real-time ultrasound imaging, the external oblique, internal oblique and transverse abdominis muscles were identified. After aseptic preparation of the injection site, a 22- gauge 10 cm insulated needle (Stimuplex A, B-Braun Medical, Melsungen, Germany) was introduced anteriorly and in the plane of the ultrasound beam until the tip was seen between the internal oblique and transverse abdominal muscles. Injectate consisted of liposomal bupivacaine 20 mL (266 mg) combined with 20 ml of 0.25% bupivacaine (total volume of 40 ml). A total of 20 ml was injected in 5 ml increments with intermittent aspiration into the fascia between the internal oblique and transverse abdominal muscles on each side. Distribution of the injectate between the internal oblique and transverse abdominis muscles was observed under real-time ultrasound imaging.

The postoperative multimodal analgesic regimen included ketorolac 30 mg, IV on arrival to the postanesthesia care unit (PACU) followed by 15 mg every 8 h and acetaminophen 1 gm PO or IV scheduled every 8 h. Ibuprofen 600 mg, orally, every 6 h was given starting on postoperative day 2. Pain scores were assessed every 2 h using a verbal numeric rating score (NRS), with 0 = no pain and 10 = worst pain. Rescue analgesics included hydromorphone 0.5 mg, IV boluses every 2 h, as needed, for severe pain (NRS > 7/10). Oxycodone 5 mg, PO, every 4 h as needed, was administered for moderate pain (NRS 4–6/10). Intravenous patient-controlled analgesia (1 mg morphine demand and lockout of 5 min) was allowed if there was significant difficulty managing pain.

Data was obtained from electronic medical records by one of the co-authors who was not involved in TAP block placement. This included patient demographics (i.e., age, ASA status, and body mass index), duration of surgery, and hospital duration. In addition, pain scores using the numeric rating scale (NRS) on arrival in the PACU, and at 2 h, 6 h, 12 h, 48 h, and 72 h after were recorded. Also, opioid consumption at the same time points as pain scores were recorded. Opioid doses were then converted to oral morphine equivalents for normalization [6].

The primary outcome measure was 48-h morphine consumption. Secondary outcome measures included interval postoperative pain scores from the PACU up to 72 h postoperatively. Sample size calculations were based on a previous study involving the use of TAP blocks in patients undergoing open abdominal surgery where the average 48-h morphine requirements were 45 mg with a standard deviation of 10 mg [7]. We considered a 25% reduction in 48-h consumption clinically significant. Based on the formula for normal theory and assuming a two-sided type 1 error of 0.05 and a power of 0.90, a minimum sample size of 21 patients per group was required. To minimize any effect of data loss, more patients were included.

**Fig. 1:**
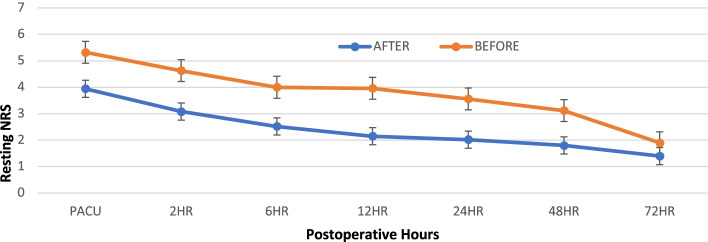
Postoperative pain scores at various time points. Data are expressed as means ± standard error. PACU = Post Anesthesia Care Unit. NRS = Numeric Rating Scale. * = *p* < *0.001*

Statistical analyses were performed using SPSS software (SPSS Inc, released 2009. PASW Statistics for Windows, Version 26.0. Chicago: SPSS Inc). Continuous data were summarized as mean ± standard deviation for normally distributed variables or as a median (interquartile range), otherwise. Baseline comparisons between the two treatment groups were made using Student’s t-test for demographic data (Table 1) or Mann–Whitney U-test (Table 2) based on the viability of normality assumption for continuous variables. The repeated measure analysis of variance and independent t-test were used to analyze differences in pain scores between groups at various points and over time (Fig. 1). Statistical significance was assessed at *p* < 0.05.

**Table 1:** Demographic/ clinical characteristics in patients undergoing emergent cesarean before and after implementation of multimodal opioid-sparing regimen

Variable	Before (*n* = 36)	After (*n* = 36)
Age, years	28.75 (6.4)	28.5 (8.1)
Height, cm	158.19 (6.5)	159.52 (5.9)
Weight, kg	81.15 (17.52)	75.82 (16.9)
Body Mass Index, kg/m^2^	32.29 (6.03)	29.54 (5.73)
Procedure Duration, minutes	59.64 (13.17)	65.5 (18.02)

Values are mean (standard deviation)

**Table 2:** Perioperative opioid consumption in patients undergoing emergent cesarean before and after implementation of multimodal opioid-sparing regimen

	**Before (*n* = 36)**	**After (*n* = 36)**
Intraoperative fentanyl (mcg)	162.5 (100, 250)	200 (100, 250)
Intraoperative hydromorphone (mg)	1.0 (0.125–2.0)	1.0 (0.45, 1.725)
IV Morphine equivalents (mg) PACU	30 (15, 38) *	1.25 (0.45, 1.725)
IV Morphine equivalents 0-24 h (mg)	99 (48, 134) *	19 (0, 32.25)
PO Morphine equivalents 0-24 h (mg)	10 (7.5, 25)^**◊**^	22.5 (7.5, 22.5)
Total Morphine equivalents 0-24 h (mg)	114 (55, 145) *	28.75 (17.5, 56.9)
Total Morphine equivalents 24-48 h (mg)	20 (10, 25) ^**◊**^	7.5 (7.5, 22.5)
Total Morphine equivalents 48-72 h (mg)	10 (5, 15) ^**◊**^	3.75 (0, 15)
TOTAL Morphine equivalents (mg)	141 (70, 185) *	65.75 (31.9, 92.5)

Values are median (interquartile range)

*PACU* Post Anesthesia Care Unit, *IQR* Interquartile Range**.** * = *p* < *0.001*, ^**◊**^ *p* < *0.01*

## Results

Table 1 shows the demographic and clinical data. Groups were comparable in terms of age, BMI, and procedure duration. There was no statistically significant difference in the amount of intraoperative opioids administered between the two groups. There was, however, a statistically significant reduction in the amount of postoperative opioid consumption in the PACU and at 24 h (*p* < 0.001), 48, and 72 h, respectively (*p* < 0.01) (Table 2). The AFTER-group showed a 25.2% reduction in morphine consumption in the first 24 h compared to the BEFORE-group and a 35.7% reduction over 48 h. In addition, total morphine equivalent requirements were significantly less in the AFTER-group compared with the BEFORE-group (*p* < 0.001) (Table 2).

There was no statistically significant difference in the pain scores in the immediate postoperative period (PACU and 2 h). However, the AFTER-group had a statistically significant reduction in pain scores at the 6 h, 12 h, 24 h and 48 h time points (*p* < 0.001) (Fig. 1).

Only 2 out of the 36 patients (5.6%) who received a multimodal opioid sparing analgesia required IV-PCA supplementation while 30 out of 36 patients (83.3%) in the BEFORE-group required IV-PCA (*p* < 0.01).

## Discussion

This quality improvement project showed that the use of a multimodal opioid-sparing pain management protocol for emergent cesarean deliveries under general anesthesia was associated with a significant reduction in postoperative opioid consumption as well as pain scores for up to 72 h.

Postoperative pain following cesarean delivery involves both somatic and visceral components, most of which involves the abdominal incision site [8]. A TAP block provides analgesia by blocking the somatic components of pain while multimodal analgesics such as NSAIDs block the visceral components [10].^.^ The multimodal opioid-sparing analgesic regimen used was a “package” that consisted of acetaminophen, an NSAID, and a TAP block. The use of acetaminophen and NSAIDs in patients undergoing cesarean delivery is well established. Although several studies have shown the efficacy of TAP blocks in patients undergoing cesarean delivery [8–10], it is not commonly used. TAP blocks are relatively simple to perform, and can be administered under general anesthesia just prior to emergence. This adds minimal time to cesarean delivery but reduces patient discomfort. Of note, a systematic review and meta-analysis by Tran et al. suggest that the use of TAP blocks for cesarean delivery do not provide additional benefits when long-acting intrathecal opioids are incorporated with multimodal analgesia [11].

Use of TAP blocks has been shown to be safe. Complications related to the TAP block can be attributed to those caused by the needle (e.g., abdominal wall hematoma and visceral injury) or systemic local anesthetic toxicity [11]. The use of ultrasound-guided TAP blocks, with the needle visualized during performance, helps to prevent the above injuries. There are several reports of systemic local anesthetic toxicity after TAP blocks when combined with neuraxial analgesia for cesarean deliveries [12–14]. Another case report of seizure was observed with repeat TAP block [15]. The risk of local anesthetic systemic toxicity is increased due to the pharmacokinetics and physiologic changes of pregnancy along with the injection of a large volume of local anesthetic [10, 12]. This can be reduced by using ultrasound guidance and constant visualization of the needle tip at the injection site with intermittent aspiration during local anesthetic injection. In addition, it is necessary to use the lowest possible local anesthetic dose necessary to achieve successful block [12].

The results of this study also demonstrate that a multimodal analgesic regimen is associated with a reduction in the need for IV-PCA opioid administration, thus reducing resource utilization. The use of IV-PCA requires the time for setup, troubleshooting, provider hand-offs and disposal of controlled substances [16]. In addition, IV-PCA use has the potential for medication errors, programming errors, and pump malfunction that could potentially compromise patient safety [16, 17]. This has led to recommendations for increased monitoring requirements for patients using PCA, including oxygen saturation and expired carbon-dioxide monitoring. Furthermore, PCA utilization requires patient and family education to ensure adequate and safe use, which adds to resource utilization. Finally, IV-PCA use restricts patient mobility due to the presence of intravenous catheters, pumps, and poles. Thus, avoidance of IV-PCA has benefits of facilitating ambulation, which is emphasized as a component of enhanced recovery after surgery [4].

The current study has several limitations. One of the limitations of this study is that it is not randomized and lacks blinding as well as contemporary controls. However, clinical quality improvement initiatives provide significant benefits including the ability to evaluate perioperative outcomes in real-world patient settings [18]. Additionally, the use of evidence-based clinical pathways in this project allowed for uniformity in patient care throughout the perioperative course including after discharge from the hospital, which improves the confidence in observed results [19]. Another limitation of this study, is that the multimodal opioid-sparing analgesic regimen used was a “package” of acetaminophen, ketorolac, ibuprofen, and TAP blocks. Therefore, the analgesic contribution of each individual component could not be defined. Also, the pain scores measured on arrival to the PACU may not reflect the contribution from TAP blocks, which were performed just prior to emergence form anesthesia. Another limitation is that our analysis only included the early postoperative period up until the third postoperative day. Follow-up after discharge from the hospital may have provided further information with respect to long-term analgesic outcomes.

In conclusion, a multimodal opioid -sparing analgesia regimen is associated with reduced postoperative opioid consumption for cesarean delivery in patients undergoing general anesthesia, and thus should be considered in the absence of neuraxial analgesia.

## Data Availability

The datasets used and/or analyzed during the current study are available from the corresponding author upon reasonable request.

## Abbreviations

NSAIDs: Non-Steroidal Anti Inflammatory Drugs
TAP: Transversus Abdominis Plane
IRB: Institutional Review Board
ASA: American Society of Anesthesiologists
IV: Intravenous
IV-PCA: Intravenous Patient Controlled Analgesia
PACU: Post-Anesthesia Care Unit
NRS: Numeric Rating Score
